# FAMILY-CENTERED COLLABORATIVE CARE FOR PATIENTS WITH CHRONIC MENTAL ILLNESS: A NARRATIVE LITERATURE REVIEW

**DOI:** 10.1192/j.eurpsy.2023.2302

**Published:** 2023-07-19

**Authors:** R. Dehbozorgi, M. Fereidooni-Moghadam, M. Shahriari, E. Moghimi-Sarani

**Affiliations:** 1 Nursing and Midwifery School, Isfahan University of Medical Sciences, Isfahan, Iran, Isfahan; 2 Department of Psychiatry Research Center For Psychiatry And Behavioral Science, Shiraz University of Medical Sciences,Shiraz, Iran, Shiraz, Iran, Islamic Republic Of

## Abstract

**Introduction:**

Chronic mental illnesses are long-lasting and recurring, require continuous care as well as an integrated and collaborative approach to organize the care. This study sought to examine whether family center collaborative care is an acceptable treatment option for individuals with chronic mental illness.

**Objectives:**

Is the family-centered collaborative care suitable for patients with chronic mental illness?

**Methods:**

From the years 2000 to 2021, ten electronic databases relating to family-centered collaborative care for mental illness were searched adopting PRISMA’s checklist

**Results:**

After systematic search, 27 articles and a thesis were found. According to moderate to high quality qualitative research, family-centered collaborative care was considered acceptable intervention, though a few studies supporting it.

**Conclusions:**

This study examines theoretical, methodological, and practical considerations as a basis for more robust data collection based on individual experiences and evidence-based practice.

**Reference(s):**

1. Wittchen, H.-U., et al., *The size and burden of mental disorders and other disorders of the brain in Europe 2010.* European neuropsychopharmacology, 2011. **21**(9): p. 655-679.
2. CUTLER, J.L., *Kaplan and Sadock’s synopsis of psychiatry.* 2016, LWW.
3. Thornicroft, G., et al., *The personal impact of schizophrenia in Europe.* Schizophrenia research, 2004. **69**(2-3): p. 125-132.
4. Vigo, D., G. Thornicroft, and R. Atun, *Estimating the true global burden of mental illness.* The Lancet Psychiatry, 2016. **3**(2): p. 171-178.

**Disclosure of Interest:**

None Declared

